# A streamlined approach for gene editing in non-obese diabetes (NOD) mice via CRISPR/Cas9

**DOI:** 10.1186/s42826-026-00281-x

**Published:** 2026-06-26

**Authors:** Kyoungin Cho, Michail S. Lionakis, Jaspal S. Khillan

**Affiliations:** 1 Mouse Genetics and Gene Modification (MGGM) Core Facility, Comparative Medicine Branch, NIAID, NIH, Bethesda, MD USA; 2 Laboratory of Clinical Immunology & Microbiology, NIAID, NIH, Bethesda, MD USA

**Keywords:** NODShiLtJ mice, CRISPR/Cas9 gene modification, Mouse embryos, Pronuclear microinjection, Polymerase chain reaction

## Abstract

**Background:**

Gene targeting via CRISPR/Cas9 has become a powerful tool to create animal models for human genetic disorders. Although CRISPR/Cas9 mediated gene editing is relatively simple, factors such as mouse strain, breeding performance, and quality and recovery of the embryos, can limit the overall outcome. Genetic manipulations are commonly carried out using embryos from a well characterized C57BL6 strain of mouse however, the ability for direct gene editing in strains such as non-obese diabetic (NOD) mice, which is an important strain to create mouse models for autoimmune type 1 diabetes (TD1), immunology, cancer and infectious diseases, is quite limiting. Therefore, gene modification in NOD mice in one way is carried out via a time-consuming cross breeding with the transgenic animals created in a more permissive C57BL6 strain for several generations which can take long time and consume valuable resources. Direct gene manipulation in the NOD mouse background will greatly expedite the creation of genetically engineered mouse models bypassing the complicated steps of backcrossing thus saving almost two years and valuable resources and help promoting 3 R principles of refinement of animal welfare.

**Results:**

We present improved and streamlined conditions for gene manipulation directly on the pure NOD background using embryos from NODShiLtJ mouse, a strain commonly used for studies on autoimmune TD1 and animal models for potential applications in cancer, immunology, and infectious disease research. High efficiency, 80–90% success rate, was achieved via CRISPR/Cas9 genome editing in pups born from embryos recovered from naturally mated 4-week-old NODShiLtJ females superovulated under specific time schedules.

**Conclusions:**

We demonstrate that 4-week-old NODShiLtJ females superovulated and mated under specific time requirements can produce healthy and robust embryos for gene manipulation saving significant resources and maintenance costs that will help in 3R principles of refinement to improve animal welfare. Similar conditions may be applied to create new genetic modifications in complex NOD/SCID Gamma (NSG) and NOD/SCID Rag (NRG) mice for dissecting mechanisms of immunology as well as generation of mouse models for infectious diseases for underlying conditions in human patients.

**Supplementary Information:**

The online version contains supplementary material available at 10.1186/s42826-026-00281-x.

## Background

Non obese diabetic (NOD) mice represent a powerful tool to study autoimmune type 1 diabetes (T1D) and insulin dependent T1D in humans [1] which, is characterized by the activation of autoreactive T cells and subsequent destruction of the insulin-producing β cells of the pancreatic Islets of Langerhans. NOD mice also exhibit multiple aberrant immunophenotypes including defective antigen presenting cell immunoregulatory functions, defects in the regulation of the T lymphocyte, defective NK cell function, defective cytokine production from macrophages and impaired wound healing [2, 3].

NODShiLtJ strain of mice is a commonly used strain for T1D, a polygenic disease with over 50 genetic linkages identified in both humans and mice which makes NOD mouse an attractive model for genetic modifications [4, 5]. This strain is also the background strain of NOD/SCID Gamma (NSG) and NOD/SCID Rag (NRG), the severely immunodeficient mice that are important for the development of next-generation human cancer and infectious disease models [6–11]. Though many variant NOD mouse models have been created representing less intense forms of T1D, improvement of the NOD as a model for human T1D by decreasing the potency of the autoimmune response remains largely unexplored. Future studies need to focus on developing multigene mouse models for detecting the diabetes susceptibility genes and their interactions that promote autoimmune diabetes. NOD mice are also useful for modeling and study autoimmune regulator disease such as AIRE deficiency and potentially important models for viral infections such as coronavirus family SARS-COV2 which is responsible for COVID19, for underlying medical conditions in human patients.

Direct gene modification in NOD mice, however, poses a significant challenge for recovery of embryos due to, a) short productive life span, b) low breeding efficiency, and c) low mating rate of the males [10, 12]. NOD mice are prone to poor health as the spontaneous autoimmunity begins at 2–3 weeks of age [13, 14] progressively targeting and destroying β-cells in the islets of Langerhans in the pancreas leading to beginning of hyperglycemia close to 12 and 15 weeks of age in females and males respectively [6, 13, 14] and are therefore, difficult to maintain for extended time periods. Furthermore, no robust NOD mouse germline competent embryonic stem (ES) cell lines are available for gene targeting. The alternate approach for creating gene modified NOD animals has relied on backcrossing with gene modified mice created on a permissive strain such as C57BL6 for several generations which, is a labor intensive and time-consuming process that can take almost 18–24 months [12]. Direct gene modification in NOD embryos, therefore, will have a significant advantage of creating mouse models on immunodeficient background in less than 3 months.

For the recovery of embryos, each strain of mouse has a different response to the hormones for superovulation [15, 16] which, requires extensive, almost 6 to 8 months of a time-consuming process to optimize conditions for hormonal regime and the timing of embryo recovery. To overcome the limitations of embryo recovery, Kumagai et al. [17] used the in vitro fertilization (IVF) approach to generate NOD embryos. The IVF, however, is a challenging procedure that involves several complicated steps and technical skills with variable success. In addition, the conditions described by this group require cumbersome hours of operation such as recovery of embryos at 2:00 AM and microinjection 15 hours later, i.e. around 11:00 PM, which may not be compatible with the time schedules for most of the laboratories as well as day and night cycle settings for mouse colonies. Here, we describe improved and streamlined conditions for the recovery of healthy and robust injectable NODShiLtJ embryos for gene modification that can reduce animal usage and colony management as well as save time and valuable resources.

CRISPR/Cas9, a highly efficient tool for gene manipulation in mouse embryos, is an adaptive immune system acquired by bacteria and Archaea for protection from the invading pathogens [18–21]. A 20nt guide RNA (gRNA) with a specific sequence in the genome is designed that binds to the target region in the chromosomal DNA after forming a complex with Cas9 protein and an 80nt tracer (transactivating CRISPR) RNA to cause double stranded break (DSB). The DSB is then repaired either by error-prone cellular repair mechanism of non-homologous end joining (NHEJ) causing insertions/deletions (InDels) or by homology directed repair (HDR) to introduce targeted mutations by a template DNA with homology arms [18–21]. Multiple genes can be targeted simultaneously with this approach [22–24].

## Methods

### Design of guide RNAs

Guide RNAs were designed using CRISPOR program http://crispor.tefor.net (Table S1). An 180 nt oligonucleotide for ACE2 gene KI was purchased from IDT inc. The synthetic gRNAs were purchased from Synthego Inc. The sgRNAs delivered as dry material were dissolved in RNase free water. The HiFi Cas9 (cat# 1081061) was purchased from IDT Inc.

### Superovulation and isolation of embryos

Eight- to 10-week-old NODShiLtJ males and 4- and 6-weeks old NODShiLtJ females were purchased from Jackson Laboratory. The males were housed individually in micro-isolator cages. All animals were maintained following the NIH ACUC guidelines under the protocol CMB15.

As shown in Fig. 3, the NODShiLtJ females were super-ovulated by injecting 5IU pregnant mare serum gonadotropin (PMSG) between 8:00 AM and 12:00 PM followed by the injection of human chorionic gonadotropin (HCG) 46 hours later. The females were then mixed with the males. The mated females with vaginal plug were separated and were sacrificed between 9:00–11:00 AM to recover the embryos.

### Microinjection of embryos

Microinjection of embryos was performed under a high power DMI 4000B Leica microscope at 100-200X magnification using continuous flow of the solution from the microinjection needle. For microinjection into pronucleus, the needle was inserted directly into one of the pronuclei to deliver the Cas9 enzyme and gRNAs which was obvious by the swelling of the pronucleus. On the other hand, for the injection into cytoplasm, the needle was inserted just below the embryo membrane to deliver the sgRNAs and Cas9 avoiding the pronuclei. At the time of isolation, the pronuclei of NODShiLtJ embryos are usually too small for microinjection. Therefore, the embryos were incubated at 37 °C for about 2-3 hr. The embryos were microinjected with sgRNA (10ng/ul) and Cas9 (5ng/ul) and oligonucleotides (2ng/ul). The surviving embryos were then transferred to the pseudo-pregnant mothers on the same day of injection to obtain pups.

### Electroporation of embryos

Electroporation of embryos is an alternate approach to microinjection into pronuclei for the delivery of CRISPR/Cas9 ingredients. Up to 150 embryos can be manipulated simultaneously using NEPA21 electroporator (NEPA GENE Co. Ltd., Japan). Since there is no insertion of needle, there is almost little or no damage to the embryos resulting in high rate of embryo survival. The embryos for the electroporation were used within 1–2 hours after isolation. The embryos with second polar body were selected under a stereo microscope at 100X magnification and electroporated inside the platinum plate electrode on slid glass (NAPA gene CO. Ltd. Japan Cat# CUY505P5) in a mixture of sgRNA (100ng/ul) and Cas9 (100ng/ul) and 200 ng/ul oligonucleotide or ssDNA using NEPA21 electroporator system (NEPAGENE Co. Ltd., Japan). The conditions for the electroporation were, 4 poring pulses at 225 V, 2.0 ms, interval 50 ms,10%voltage decay and polarity+ followed by 5 transfer pulses of 20 V, 50 ms, interval 50 ms, 40% voltage decay transfer pulses of 20 V, with alternating and polarity. The Impedance was measured and maintained between 0.490 and 0.509 kΩ by liquid volume adjustment. The embryos were then transferred to M2 medium (MilliporeSigma, USA, cat# MR015) to wash out the RNP complexes then incubated in KSOM medium (MilliporeSigma, USA, cat# MR101) at 37 °C, 5% CO2 until the oviduct implant procedure.

**Pronuclear microinjection vs Electroporation of embryos**: Pronuclear microinjection of embryos is performed via direct delivery of ingredients into the pronucleus and is a highly reliable procedure for the injection of small nucleotides to large plasmids [16]. Only a small amount of the injection material is required for microinjection. However, only a limited number of embryos can be microinjected in any given session, and the viability rate after microinjection is lower than electroporation due to the physical injury caused by the needle. Microinjection requires highly trained personnel, maintenance of the equipment and labor intensively. The success rate of genome modification can vary between 10 and 50% of the liveborn pups. On the other hand, the electroporation is relatively simple procedure in which the embryos are placed between the two electroplates in a medium carrying Cas9 enzyme, sgRNAs and oligonucleotides. The survival rate of embryos is almost 100% which results in a high pup rate. The success rate of gene modification can vary between 30 and 80%. However, the limitations of this procedure are that large plasmids or large DNA fragments cannot be electroporated. Further, it requires large amounts of injectables which adds to the high costs.

### Preparation of pseudo-pregnant mothers

Five to 6-weeks old CD1 females were mated with the vasectomized CD1 males [16]. The females with the vaginal plug were separated as pseudo-pregnant mothers. The electroporated or microinjected embryos were surgically transferred into the oviducts of pseudo-pregnant mothers to generate pups.

### Screening of animals

After the weaning, total DNA isolated from ear punch samples from the pups were analyzed by PCR amplification using specific primers under denaturation at 95 °C 5 mins; (95 °C 30 sec, 60 °C 30 sec, 72 °C 30 sec) ×27 cycles and 72 °C 3 mins conditions. The amplified DNA fragments were separated on 2% agarose gels and visualized in Azure imager (AZURE BIOSYSTEM – Bioanalytical Imagining System).

### Breeding of lines and sequencing of DNA

Gene modified males at 8-week and females at 6-week were crossed with wild type animals to obtain the progeny. The DNA from the F1 generation pups was sequenced by Sanger sequencing to confirm the genome editing. The animals with correct gene KI insertion were selected for breeding and establishing the lines. The gene targeted animals were bred with wild type animals for at least three generations for germline transmission and elimination of off-site targets of the mutation.

## Results

### Design of sgRNA

**Fig. 1:**
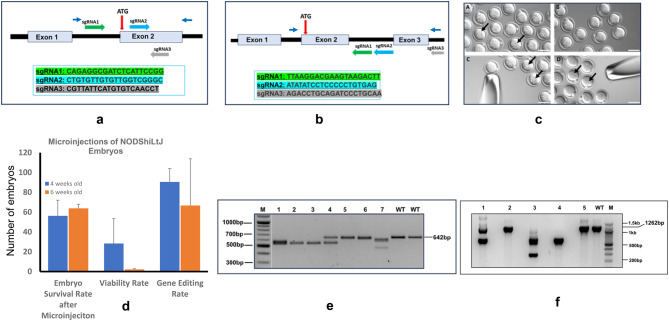
Guide RNAs, embryo collection and screening. **a** Gasdermin D gene KO: top panel shows the position of the gRnas used for the Gasdermin D gene KO. Bottom panel shows the sequence of each gRNA. The gRnas are designed to create deletion in intron 1 and exon 2 of Gasdermin D including ATG codon. **b**. Trpm5 gene KO: top panel shows the position of the gRnas used for the gene KO. Bottom panel shows the sequence of each gRNA. The gRnas are designed to create deletion of the exon 3 of Trpm5. Blue arrows in each figure shows the location of the primers for Gasdermin D and Trpm5 genes, respectably. **c**. Injectable embryos from NOD mice: panel a & C, C57BL6/J mouse embryos post 72 hours PMSG showing prominent pronuclei (marked by arrows). Panel B, NOD mouse embryos post 72 hours PMSG do not show clear pronuclei for microinjection. Panel D: NOD mouse embryos post 77 hours PMSG showing prominent and clear pronuclei (marked by arrows). Bars = 50 µm. **d**. The comparison of microinjection between 4-week and 6-week-old NODShiLtJ mice. Embryo survival rate was calculated by dividing the number of viable embryos after the microinjections by total number of microinjected ones. Viability rate was calculated by dividing the number of live pups by total number of implanted embryos. Gene editing rate was calculated by dividing the number of CRIPR edited animals by total number of live pups. ns: non-significant, *n* = 3, paired t-test. **e**. Polymerase chain reaction (PCR) screening of animals. a. Screening of Gasdermin D pups. Samples #1, #2, #3, #4 and #7 amplify bands smaller than the normal wild-type (WT) band of 642bp. **f**. screening of Trpm5 pups. Samples #1, #3 and #4 amplify bands smaller than the normal wild-type (WT) band of 1262bp

To validate the gene manipulation in NODShiLtJ mouse embryos, we targeted three independent genes such as, gene knockout (KO) of Gasdermin D [25, 26], gene KO of transient receptor potential cation channel sub family member 5 (Trpm5) [27], and the gene knock-in (KI) of angiotensin converting enzyme 2 (ACE2), a receptor for SARS-COV-2 corona virus [28]. We used CRISPOR program (https://crispor.gi.ucsc.edu) to design the gRNAs. More specifically, the gene KO gRNAs for Gasdermin D and Trpm5 were designed to introduce a deletion of an exon containing the initiation codon (Gasdermin D) or a complete exon (Trpm5) to cause total inactivation of the gene (Table S1, Fig. 1, panel a and b). The sgRNAs were selected based on MIT score with lowest offsite targets assigned by the CRISPOR software to ensure high on-target activity. Any gRNAs with off-target location within 20Mb in the same chromosome were eliminated to prevent possible genetic linkage with the desired mutation to exlude the necessity for off-site target analysis. Irrespective of that, the animals were bred for at least three generations to eliminate any inadvertent off target event.

### Recovery of NODShiLtJ embryos

Though in vitro fertilization (IVF) using sperm and oocytes, is an alternate approach to create large number of embryos, the embryos recovered from natural mating of males and females are generally healthy and robust for gene manipulation [16]. The recovery of embryos is highly dependent of the strain and age of the donor females. Natural mating of NOD mice, however, is not very efficient and only small percentage of animals mate resulting in low yield of fertilized embryos [17]. Therefore, to determine the appropriate age of NODShiLtJ donors, females at 4-weeks and 6-weeks were used for superovulation. Table 1 summarizes the superovulation response from three independent sets of 4-week and 6-week-old NODShiLtJ females. As shown in the table, 6-week-old females consistently exhibited higher rates of mating compared to 4-week-old females suggesting that older NODShiLtJ females are more compatible and permissive for mating compared to younger females. Further, the yield of total oocytes from 4-week-old females is significantly lower than the 6-week-old females supporting the earlier studies [11] with NOD/SCID mice. Regardless, we found no significant difference in the average number of injectable embryos per female, i.e. 22 embryos from 4-week and 24 embryos from 6-week-old suggesting that 4-week-old females are suitable for embryo recovery that can save time and costs required for an additional two weeks of maintenance of mouse colony.

**Table 1:** Superovulation response of NODShiLtJ mice

Age of females	# of females	# of females plugged	# of embryos	# of healthy Injectable embryos	Average # of injectable embryos/female
4 weeks	5 5 5	3 2 4	124 94 133	75 28 94	22
Total	15	9	351	197	
6 weeks	5 5 5	5 5 4	175 135 155	137 98 96	24
Total	15	14	465	331	

### Pronuclear injection of embryos

The embryos were first screened under a stereo microscope for the morphology of the pronuclei. It was observed that at the time of embryo isolation, the pronuclei of the embryos were very small and not clearly visible for microinjection (Fig. 1c) suggesting the slow development of NODShiLtJ embryos. Earlier reports on microinjection of in vitro fertilized NOD/SCID mouse embryos after 8 hours of IVF did not produce any pups, however, the efficiency of production of pups improved to ~27% after the microinjection was delayed by 15 hours after IVF [17]. Therefore, we incubated the embryos for 2–3 hours before microinjection with sgRNA and Cas9, i.e. approximately at 76 hours of PMSG injection.

A total of 197 embryos and 331 embryos were injected from 4-wk-old and 6-wk-old females respectively. An approximately similar percentage of embryos between the two age groups, 118 (59.8%) for 4-week- and 213 (60.6%) 6-week-old survived after microinjection, then transferred into the oviducts of pseudo-pregnant females to obtain pups. A total of 18 pups, and 5 pups were born from 4-week-old and 6-week-old females respectively (Supplementary table S2). The pups were screened by PCR using primers that amplify a 642bp fragment from normal wild type (WT) allele. Figure 1eshows representative samples from 7 pups. As shown in the figure, five pups shown to carry deletion of the Gasdermin D gene. A total of 16 pups out of 18 alive pups (88.8%) from 4 weeks old embryo donor and 4 out of 5 (80%) from 6 weeks old donor respectively, were positive for the gene deletion suggesting there is no statistical difference with the gene editing rate among live pups between two groups (Fig. 1d). However, the number of gene edited animals per donor female in 4 weeks old group is significantly higher than that of 6 weeks old group (Supplementary Figure 1, 10.06 $\pm$ 0.30 vs 0.26 $\pm$ 0.03, *p* = 0.032) Overall, these results suggest that embryos from 4-week-old NODShiLtJ females are more robust and are suitable for gene manipulation.

Subsequently, two sets of NODShiLtJ embryos from 4-week-old females were microinjected using two sets of guide RNAs, i.e. #1and #3 or #2 and #3 (Table 2), for the Trpm5 gene into 75 and 81 embryos respectively (Table 2A). The pups were screened by PCR using primers that amplify a 1262bp fragment. Figure 1fshows PCR results from five representative samples. A total of 12 pups from first set of #1 and #3 guide RNAs, and 2 pups from second set of #2 and #3 guide RNAs were born of which, 11 pups (91.66%) and 1 pup (50%) respectively, were positive for the gene deletion. Overall, the microinjection of 123 embryos in these two sets showed gene deletion in 86% of the pups (Table 2A).

**Table 2:** Trpm5 gene knockout of NODShiLtJ embryos

**A**: Pronuclear microinjection of NODShiLtJ embryos						
**Microinjection **	**# of embryos **	**sgRNA #**	**#survived**	**#of pups**	**#of positive pups**	**% positive pups**
1	75	*#1r+#3r*	65	12	11	91.66
2	81	#2r+#3r	58	2	1	50
**Overall positive **				14	12	86
**B**: Electroporation of NODShiLtJ embryos						
**Electroporation**	**# of embryos**	**sgRNA #**	**#survived**	**#of pups**	**#of positive pups**	**% positive pups**
1	63	*#1r+#3r*	61	7	6	85.7
2	53	*#2r+#3r*	53	4	3	75
**Overall positive **				25	21	84
**C**: Summary of Pronuclear microinjection and electroporation of NODShiLtJ embryos						
**Expt. #**	**Method **	**# of embryos electroporated**	**#survived**	**#of pups**	**#of positive pups**	**% positive pups**
1	Microinjection	156	123	14	12	86
2	Electroporation	116	114	11	9	81

### Electroporation of embryos

Electroporation of sgRNA and Cas9 into embryos has been used as an alternative to microinjection for successful CRISPR/cas9 gene targeting [29–32]. As described above, large number of embryos can be manipulated simultaneously via electroporation. The embryos for electroporation were manually selected under the microscope based on the appearance of the second polar body.

A set of 140 NODShiLtJ embryos were electroporated with a mixture of Gasdermin D sgRNA and Cas9 enzyme. A total of 138 embryos that survived the electroporation was transferred to the foster mothers which produced 54 pups. The screening of ear punch samples revealed that only 1 pup exhibited targeting of the gene (Table S3) suggesting that targeting of Gasdermin D gene is more efficient by direct microinjection into the pronucleus.

To test further, two separate sets of embryos were electroporated with two sets of Trpm5 sgRNAs described above (Table 2B). The pups were screened by PCR using primers (Fig. 1b) that amplify a 1262 bp fragment as described above (Fig. 1f). As shown in table 2B, the first set of 63 embryos electroporated with sgRNA #1 and #3 produced 7 pups of which, 6 pups (85.7%) exhibited deletion of the gene, whereas the second set of electroporation of 53 embryos with sgRNA #2 and #3 generated 4 pups of which 3 pups (75%) were positive for the deletion (See Table 2C).

**Fig. 2:**
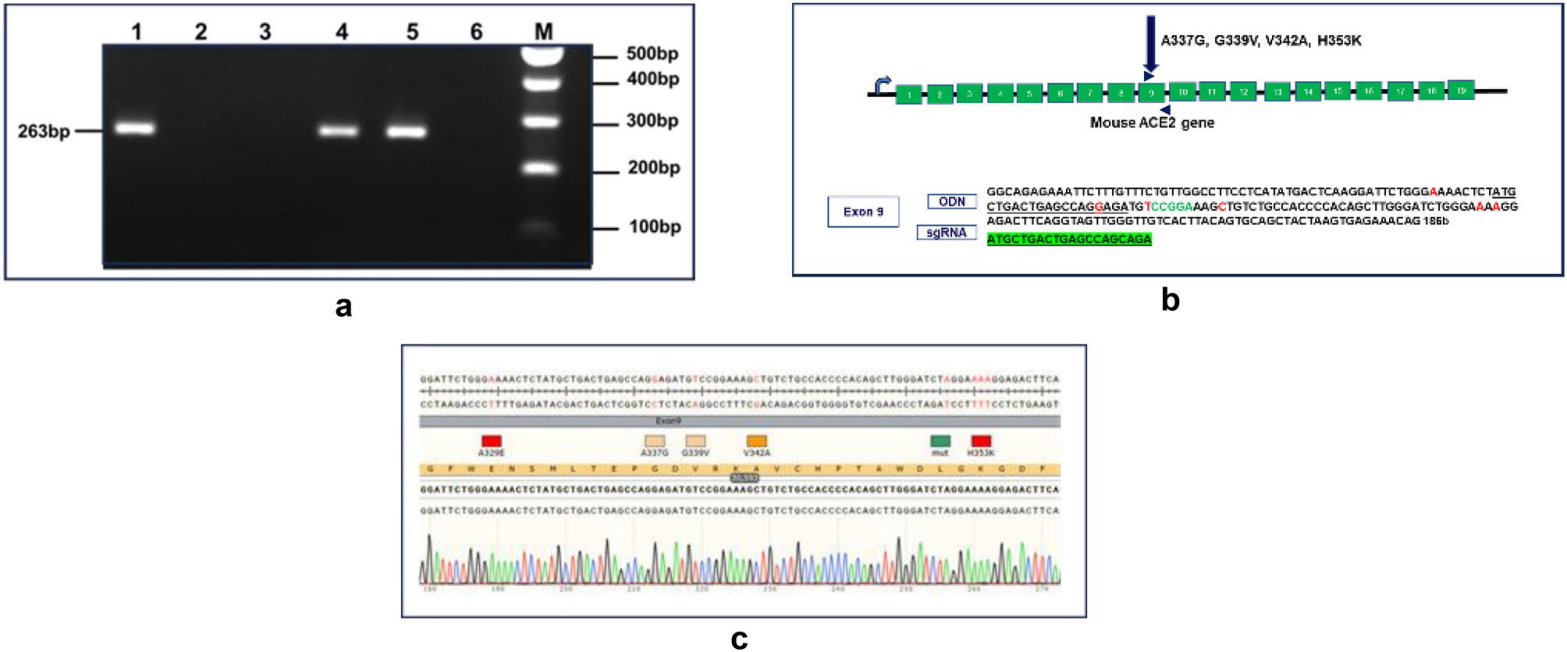
Screening of ACE2 pups. **a**. Samples #1, #4 and #5 amplify an expected 263bp band. No amplification is observed in negative samples. **b** structure and organization of exons of ACE2 gene and the Sanger sequencing. **a**. upper panel, showing the exon structure of the gene and the targeted amino acids in exon9. lower pane, the sequence of gRNA and 186b oligonucleotide. The position of gRNA in ODN is underlined and the targeted nucleotides corresponding to specific amino acids are shown in color. **c**. Sanger sequencing of DNA from ACE2 gene targeted animals: a snapshot of sequence alignment between CRISPR template and amplified region around mouse exon9 from a male hACE2 mice, demonstrating the gene edition in exon 9 of the mouse ACE2 gene. Altered nucleotides were shown in red, converting the designated codon into the human ortholog amino acid

Next, we tested the electroporation strategy to target the ACE2 gene, a receptor for SARS-COV-2 corona virus [28], by substituting five mouse amino acids with human amino acids, i.e. A329E, A337G, G339V, V342A and H353K in exon 9, using a sgRNA along with either a 533nt single stranded DNA (ssDNA) with large homology arms or 186nt oligonucleotide (Fig. 2b). Table 3 shows the electroporation of three sets of embryos using sgRNA/Cas9 along with either ssDNA or the oligonucleotide. The DNA from pups was screened using primers that amplify 263bp of the genomic sequence (Fig. 2a). Electroporation of two sets of embryos, 87 and 30, produced 10 and 3 pups respectively with ssDNA of which 3 pups (30%) and 1 pup (33%) were positive for the correct gene KI. On the other hand, the electroporation of oligonucleotide into 101 embryos generated 8 pups of which only one pup (12.5%) was positive for the correct gene KI indicating that both oligonucleotides and ssDNA can be used effectively for the gene KI modification in NOD embryo though the gene KI via ssDNA was more efficient (Table 3) supporting previous studies [33]. The correct gene insertion was further confirmed by Sanger sequencing (Fig. 2c).

**Table 3:** Gene targeting of mouse ACE2 exon 9 mutation

Expt. #	# of embryos	# survived	# of pups	# of positive pups	Homology Template	% positive pups
Electroporation	87	72	10	3	ssDNA	30
	30	17	3	1	ssDNA	33
	101	72	8	1	oligo	12.5

### Cytoplasmic injection of embryos

Since direct microinjection into the pronucleus has its limitations, microinjection into the cytoplasm has been used as an alternative strategy whereby many embryos can be injected simultaneously, and the rate of embryo survival is also high. On the other hand, microinjection into the cytoplasm is known to be very inefficient for gene modification [34, 35]. To test this approach for NODShiLtJ embryos, a mixture of Gasdermin D sgRNA and Cas9 was microinjected into the cytoplasm of the embryos. As shown in Table 4, the microinjection of 206 embryos produced 26 pups. Screening of the ear punch DNA by PCR revealed that none of the pups exhibited the targeting of the Gasdermin D gene, confirming the inefficiency of cytoplasmic microinjection [34, 35] which may be attributed to the non-permissive nature of the nuclear membrane for diffusion of sgRNA/Cas9 complex.

### Breeding and germline transmission

To test germline transmission and propagation of lines, the gene targeted males at 8 week and females at 6 weeks were crossed individually with wild-type counterparts. The progeny was then analyzed by PCR following the strategy shown in Figs. 1a, 1b and 2b. The progeny from all the three targeted genes carried the modified gene confirming the successful germline transmission. The progeny of the ACE2 animals was further confirmed by Sanger sequence similar to as shown in Fig. 2c.

### Establishment of mouse colonies

The gene modified animals for all the genes were bred for at least three generations to eliminate any potential off-site targets [36]. The gene modified Gasdermin D and Trpm5 mice were further screened for the expression of protein (Data not shown). None of the mice showed expression of the protein proving complete inactivation of the gene. The gene modified males and females were then crossed with each other to create homozygous animals which was followed by cryopreservation of the embryos and sperm for long term storage and to prevent the genetic drift.

## Discussion

Genetically engineered animals play a critical role in the basic and clinical research for understanding the underlying mechanisms that govern various medical conditions. Mouse is the most convenient model for gene manipulation for gaining the basic knowledge which can be used for clinical applications [37]. High precision targeting efficiency of the CRISPR/Cas9 technology has become a fast approach for the creation of gene modified animals quickly [18–21]. Although the CRISPR/Cas9 technology has significantly improved the efficiency of generating mouse models, the limiting step however, is the accessibility of healthy and injectable embryo.

Embryos from mouse strains such as C57BL6, FVB/N, Swiss webster, Balb/C, 129 SV and NOD have been manipulated to create gene modified animals, however, each strain exhibits different hormonal response for the recovery of embryos, morphological features of the embryos and in vitro culture conditions [15, 16]. Therefore, optimal conditions need to be developed for each strain for the recovery of embryos reproducibly.

The inbred C57BL6 mouse stain is the most used strain for gene manipulation because it has a well characterized genome and is highly inbred [38]. NOD mice, on the other hand, represent a valuable model system to study autoimmune T1D - and insulin dependent diabetes mellitus (IDDM) in humans. Further, the NOD/SCID Gamma (NSG) and NOD/SCID Rag (NRG), created by gene manipulation of NODShiLtJ mouse embryos, have been used extensively for immune disorders, cancer and cell engraftment studies [39]. Further gene modification in these strains, therefore, can be valuable to generate improved models for human diseases and enhanced cell transplantation studies. The approach of backcrossing these mice with transgenic animals can be very complicated and time-consuming due to the complexity of their genetic background. Furthermore, these strains do not respond well to hormone treatment and therefore, natural breeding is preferred for the recovery of embryos for gene manipulation [10]. The NOD mice are also potentially useful models to investigate infections such as those of coronavirus family SARS-COV1, MERS and SARS-COV2 which is responsible for the recent COVID-19 pandemic for underlying conditions in human patients.

As reported earlier, it is challenging to obtain fertilized eggs from mice with NOD background via natural mating, therefore, an alternate approach, of in vitro fertilization (IVF) has been used to obtain embryos [11, 17]. Mating of 8-week-old NOD/SCID females with 8–12-week-old males resulted in significantly lower efficiency of mating (13.3%) with only 6% of fertilized embryos, however, a high yield of embryos could be achieved via IVF [17]. IVF is a widely used assisted reproduction technology; however, it is complex and challenging procedure which requires specialized equipment and highly skilled workers and involves additional labor-intensive complex and time-consuming steps. Kumagai et al. [17] used a complex time schedule for superovulation to recover NOD/SCID oocytes for IVF which can be limited for regular laboratory situations [17]. However, Li et al. [11] on the other hand, were able to recover embryos from 4 to 10-week NRG females super ovulated by 5IU PMSG and 48 hour later 5IU HCG followed by IVF.

**Fig. 3:**
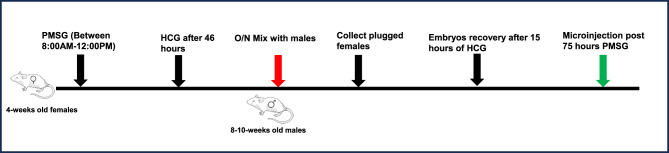
Timeline for the recovery of NOD embryos and manipulation: 4-weeks old females are injected with PMSG between 8:00AM and 12:00PM. HCG is injected after 46 hours, and the females are mixed with the males, and the plugged females are collected next morning. The embryos are recovered after 15 hours of PMSG and microinjected at 75 hours post PMSG

Microinjection of in vitro fertilized NODSCID embryos after 8 hours of IVF did not produce any pups leading to the speculation that microinjection of embryos with significantly small and poorly visible pronuclei may have led to the damage to the zygotes resulting in the failure to develop normally [17]. The efficiency of the production of pups from IVF derived embryos, therefore, improved to approximately 27% after the microinjection was delayed by 15 hours [17]. Previous studies have shown that the embryos from some strains develop slower, however, their pronuclei become more prominent after 16–20 hours post fertilization [15, 16]. Lin et al. [40] previously reported the creation of *Ptpn22*^*R619W*^ mutant mice by microinjection into NODShiLtJ embryos isolated from 12-week-old superovulated females. However, the detailed procedures for manipulation of embryos are unavailable. A CARD Hyperova reagent (Cosmo Bio USA) has been used successfully for superovulation of 12-week-old NSG mice (Personal communication from Yun You, University of Minnesota, mouse Genetic Laboratory). Simplified procedure using younger mice with the exclusion of complex time schedules and additional procedures such as IVF will save significant amount of time, animal costs and valuable resources. Here we demonstrate that younger 4-week-old NODShiLtJ females, under modified time schedules can generate robust and healthy embryos for efficient gene manipulation. Our studies reveal that 4-week-old females superovulated by PMSG injection between 8:00 AM-12:00 PM followed by injection of HCG after 46 hours then mated immediately with the stud males followed by recovery of embryos after 15 hours of mating is highly efficient for robust and healthy injectable embryos (Fig. 3). Despite a low mating rate in 4-week-old females compared to 6-week-old females, no significant difference was observed in the average number of injectable embryos (Table 1). Moreover, embryos from the 4-week-old females under this superovulation regime resulted in significantly higher number of gene modified pups per female that were used in total (Figure S1). Using 4-week-old mice, therefore, can save significant amount of maintenance costs and efforts of the investigators. Though in a limited study, Kim et al. [41]) has also reported successful genome modification using embryos from 3 to 4-week-old NODShiLtJ mice, 5IU PMSG and 48 hour later HCG superovulation, using Cpf1 enzyme, the current studies however, provide a detailed analysis of embryo recovery and embryo survival under different conditions using most commonly used Cas9 enzyme.

To validate the conditions for gene manipulation, we have tested several parameters such as a) gene KO mutations, b) direct microinjection into the pronucleus, c) microinjection into the cytoplasm, d) electroporation and e) gene KI via oligo nucleotide and single stranded DNA. Our studies show that successful gene modification in NODShiLtJ embryos can be achieved by direct microinjection into the pronucleus as well by electroporation. Though successful gene insertion could be achieved with ssDNA as well as oligonucleotides (Table 3), the success rate is much higher with the ssDNA which carries longer homology arms in agreement with the previous studies [33].

Direct delivery of foreign genetic materials into the pronucleus of embryo is the most efficient approach for creating genetically engineered mouse models. However, the embryos from NOD mice, are difficult for pronuclear microinjections due to the slow growth of the embryo and poor visibility of the pronuclei (Fig. 1c) which can be overcome by culture of embryos for few hours. Direct microinjection of CRISPR materials into the pronucleus of NODShiLtJ embryos resulted in more than 85% gene modified pups (Tables S2 and 2C). One drawback, however, is the low pup number due to damage to the embryos caused by the microinjection. On the other hand, it is important to note that the targeting efficiency may vary from gene to gene and the sgRNAs used. Similar efficiency of gene targeting can be achieved with either direct pronuclear microinjection or electroporation (Table 2C). Cytoplasmic injection on the other hand, failed to generate any pups with gene modification (Table 4) which has very limited success especially with the plasmids and large gene constructs [34, 35]. Successful injection of large plasmids or DNA sequences e.g. 10-15kb size or Bac clones of >150kb, however, can be performed only by direct microinjection into the pronucleus [42].

**Table 4:** Cytoplasm microinjection of GasderminD sgRNA

	Expt. #	# of embryos	# injected	# survived	# of pups	# of positive pups
**Cytoplasm microinjection**	1 2	130 200	86 120	68 90	9 17	0 0
	Total	330	206	158	26	0

Electroporation of sgRNA, Cas9 and oligonucleotides into embryos also showed a high success rate of genome modification (Tables 2B and 3). Since the zona pellucida of the embryos is a strong barrier for entry of any material, a brief treatment with acidic Tyrode’s solution was suggested to soften the zona pellucida [30–32]. However, subsequent studies [31] and our current studies have revealed that this treatment is unnecessary.

Overall, we present simple and streamlined conditions for direct gene manipulation on pure NOD background without requiring the lengthy and time-consuming process of backcrossing transgenic animals, which can save significant resources, time and usage of animals. We present a detailed analysis of the comparison of number of animals and quality of embryos from different age groups not reported earlier and validating using multiple genes and most used Cas9 enzyme. In addition, we show that modified superovulation regime of 46-hour gap between PMSG and HCG is equally successful for creating injectable embryos from 4-week-old females with high success rate of gene modification per female mouse.

## Conclusions

Direct microinjection of genetic materials into the pronucleus or electroporation into embryos from 4-week-old NOD mice is a high efficiency approach for gene modification on the pure NOD mouse background bypassing the need for lengthy backcrossing for several generations and allowing for saving of time, efforts and valuable resources as well as supporting the 3 R principles of refinement of animal welfare. Using CRISPR/Cas9 genome editing, high percentage gene modified animals on pure NOD background could be generated in approximately 12–14 weeks.

## Electronic supplementary material

Below is the link to the electronic supplementary material.

Supplementary Material 1

## Data Availability

The data will be provided to the investigators upon request.

## Declarations

### Animal welfare

These studies will help in reducing the number of animals used, unnecessary prolonged colony maintenance, avoiding backcrossing for over 12–18 months thus ultimately helping the goal of 3 R principles of refinement to improve animal welfare.

## Abbreviations

ACE2: Actinomycin converting enzyme 2
HCG: Human chorionic gonadotropin
NOD: Non-obese diabetic
PCR: Polymerase chain reaction
PMSG: Pregnant mare serum gonadotropin
ssDNA: Single stranded DNA
WT: Wild type
