# Supplementary material for: A streamlined approach for gene editing in non-obese diabetes (NOD) mice via CRISPR/Cas9
Source: Lab Anim Res. 2026 Jun 26;42:24. doi: 10.1186/s42826-026-00281-x (PMC13308463; doi:10.1186/s42826-026-00281-x)
Supplement: Supplementary file 1 — Supplementary Material 1 [file 42826_2026_281_MOESM1_ESM.docx]

**Table S1**: The gRNAs used for the gene editing of Gasdermin D, Trpm5 and ACE2.

| **Gene** | **sgRNA** | **Sequence** | **MIT score** (Computed by the CRISPOR web tool) |
| --- | --- | --- | --- |
| Gasdermin D | #1  #2  #3 | CAGAGGCGATCTCATTCCGG  CTGTGTTGTGTTGGTCGGGC  AGGTTGACACATGAATAACG | 91  88  81 |
| Trpm5 | #1  #2  #3 | TTCAGAATGAAGCAGGAATT  GAGTGTCCCCCTCCTATATA  AACGTCCCTAGACGTCCAGA | 75  80  71 |
| ACE2 | #1 | ATGCTGACTGAGCCAGCAGA | 59 |

**Table S2:** Microinjection of NODShiLtJ embryos

| **Age of females** | **# of embryos** | **# of healthy embryos/**  **Microinjected** | **# survived**  **(%survival)** | **# of pups** | **# of gene targeted pups (%)** |
| --- | --- | --- | --- | --- | --- |
| 4 weeks | 124  94  133 | 75  28  94 | 58  11  49 | 4  7  7 | 4  5  7 |
| Total | 351 | 197 | 118 (58.6) | 18 | 16 (88.8) |
| 6 weeks | 175  135  155 | 137  98  96 | 94  63  56 | 3  1  1 | 3  1  0 |
| Total | 465 | 331 | 213 (60.6) | 5 | 4 (80.0) |

**Table S3:** Electroporation of GasderminD sgRNAs into NODShiLtJ embryos

| **Age of females** | **# of females** | **# of females plugged** | **# of embryos** | **# of healthy embryos** | **# survived** | **# of pups** | **# of gene targeted pups (%)** |
| --- | --- | --- | --- | --- | --- | --- | --- |
| 6 weeks | 10 | 8 | 290 | 140 | 138 | 54 | 1 (1.85) |


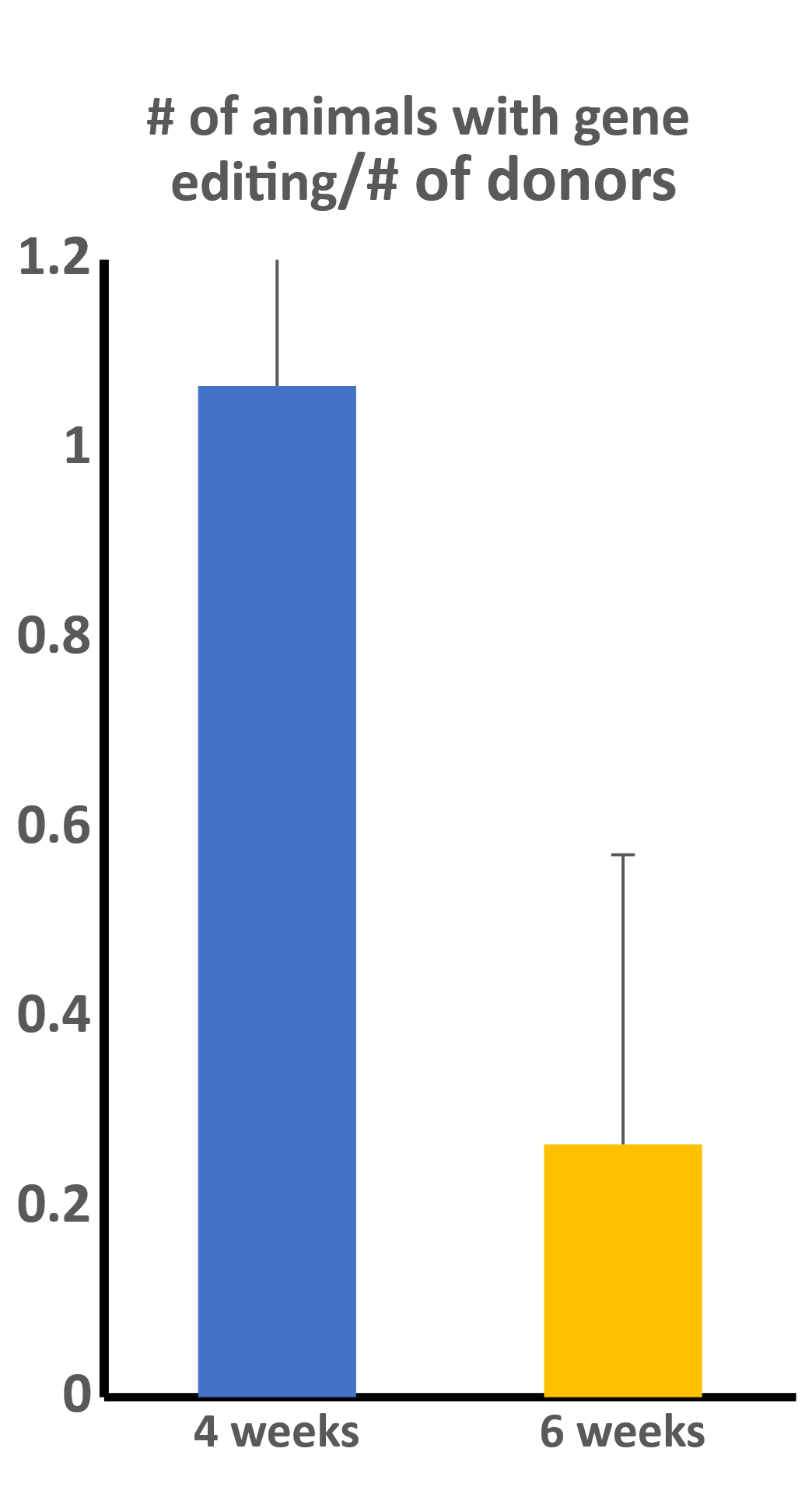


**Figure S1**.Gene editing efficiency shown by the number of animals with gene edition per each female donor group, 4- and 6-weeks old females. The comparative gene editing efficiency in embryos from 4 week and 6-week-old females reveal significant advantage for embryos from 4wk old females in CRISPR gene targeting of NOD strain.
